# Respiratory motion compensation in interventional liver SPECT using simultaneous fluoroscopic and nuclear imaging

**DOI:** 10.1002/mp.13653

**Published:** 2019-06-27

**Authors:** Martijn M. A. Dietze, Remco Bastiaannet, Britt Kunnen, Sandra van der Velden, Marnix G. E. H. Lam, Max A. Viergever, Hugo W. A. M. de Jong

**Affiliations:** ^1^ Radiology and Nuclear Medicine Utrecht University and University Medical Center Utrecht P.O. Box 85500 3508 GA Utrecht the Netherlands; ^2^ Image Sciences Institute Utrecht University and University Medical Center Utrecht P.O. Box 85500 3508 GA Utrecht the Netherlands

**Keywords:** motion compensation, radioembolization, SPECT

## Abstract

**Purpose:**

Quantitative accuracy of the single photon emission computed tomography (SPECT) reconstruction of the pretreatment procedure of liver radioembolization is crucial for dosimetry; visual quality is important for detecting doses deposited outside the planned treatment volume. Quantitative accuracy is limited by respiratory motion. Conventional gating eliminates motion by count rejection but increases noise, which degrades the visual reconstruction quality. Motion compensation using all counts can be performed if the motion signal and motion vector field over time are known. The measurement of the motion signal of a patient currently requires a device (such as a respiratory belt) attached to the patient, which complicates the acquisition. The motion vector field is generally extracted from a previously acquired four‐dimensional scan and can differ from the motion in the scan performed during the intervention. The simultaneous acquisition of fluoroscopic and nuclear projections can be used to obtain both the motion vector field and the projections of the corresponding (moving) activity distribution. This eliminates the need for devices attached to the patient and provides an accurate motion vector field for SPECT reconstruction. Our approach to motion compensation would primarily be beneficial for interventional SPECT because the time‐critical setting requires fast scans and no inconvenience of an external apparatus. The purpose of this work is to evaluate the performance of the motion compensation approach for interventional liver SPECT by means of simulations.

**Methods:**

Nuclear and fluoroscopic projections of a realistic digital human phantom with respiratory motion were generated using fast Monte Carlo simulators. Fluoroscopic projections were sampled at 1–5 Hz. Nuclear data were acquired continuously in list mode. The motion signal was extracted from the fluoroscopic projections by calculating the center‐of‐mass, which was then used to assign each photon to a corresponding motion bin. The fluoroscopic projections were reconstructed per bin and coregistered, resulting in a motion vector field that was used in the SPECT reconstruction. The influence of breathing patterns, fluoroscopic imaging dose, sampling rate, number of bins, and scanning time was studied. In addition, the motion compensation method was compared with conventional gating to evaluate the detectability of spheres with varying uptake ratios.

**Results:**

The liver motion signal was accurately extracted from the fluoroscopic projections, provided the motion was stable in amplitude and the sampling rate was greater than 2 Hz. The minimum total fluoroscopic dose for the proposed method to function in a 5‐min scan was 10 µGy. Although conventional gating improved the quantitative reconstruction accuracy, substantial background noise was observed in the short scans because of the limited counts available. The proposed method similarly improved the quantitative accuracy, but generated reconstructions with higher visual quality. The proposed method provided better visualization of low‐contrast features than when using gating.

**Conclusion:**

The proposed motion compensation method has the potential to improve SPECT reconstruction quality. The method eliminates the need for external devices to measure the motion signal and generates an accurate motion vector field for reconstruction. A minimal increase in the fluoroscopic dose is required to substantially improve the results, paving the way for clinical use.

## 1 Introduction

Respiratory motion is a major degrading factor for the quantitative accuracy of single photon emission computed tomography (SPECT) scans and should ideally be compensated for in the reconstruction.^1^, ^2^, ^3^ Clinically, the effect of motion is mitigated by applying gating: a projection set is generated by only incorporating data with a minimum motion amplitude.^4^ This improves the resolution but increases the noise owing to the limited counts that are accepted, especially when performing fast scans. More elaborate methods that use all data additionally require a motion vector field: a map of the patient‐specific organ movements and deformations over the respiratory cycle.^5^ Using such a model, the data from all individual motion phases can be combined into a single motion‐corrected reconstruction.^6^

To extract the motion signal, data‐driven approaches are often used in other modalities.^7^, ^8^ However, since SPECT scans are often limited by count statistics, data‐driven approaches are more challenging for this modality^9^ and additional information is usually required. For instance, the respiratory signal can be tracked using a respiratory belt, which measures the expansion of the abdomen over time. Other frequently used approaches include camera systems and spirometers.^10^ Most of these devices require extra work for the technicians and are difficult to work with in time‐critical situations.

It is difficult to construct a general model for the motion vector field due to interpatient variability and different breathing preferences.^11^ Therefore, the most common approach to estimate the motion vector field is to extract it per patient from a previously acquired four‐dimensional (4D) scan, such as computed tomography (CT)^12^ or magnetic resonance imaging (MRI).^13^ However, observed motion can depend appreciably on the scan modality or procedure,^14^ for example, because of different levels of stress or scan duration. Hence, the motion vector field from these scans potentially describes the motion in the scan of interest suboptimally.

A device that simultaneously measures nuclear and fluoroscopic projections,^15^, ^16^ and that is now under construction, could be used to intrinsically correct for respiratory motion. The motion signal can be extracted from the fluoroscopic projections and be used to allocate the nuclear and fluoroscopic projections to motion bins. The fluoroscopic projections are reconstructed per bin and then registered to each other to extract the motion vector field over time, which can subsequently be included in the SPECT reconstruction.^17^ A single, averaged motion vector field is thus used to correct the data over the entire scan. The advantages of such a device are twofold: no external devices are required to extract the motion signal, simplifying the procedure in the clinic, and the motion vector field is retrieved from the same scan, ensuring that changes in breathing between several scans do not influence motion compensation performance. The disadvantages are that gamma sensitivity is reduced and extra dose is administered.

Our approach to motion compensation will most likely not be suitable for general motion compensation, but there are situations in which it could be of benefit. The proposed use for our device is in hepatic radioembolization, in which small radioactive microspheres are inserted into the liver.^18^ A pretreatment SPECT/CT scan of technetium‐99m macroaggregated albumin is normally performed before therapy to detect potential inadvertent regions of high activity and for the treatment planning of dosimetry. Ideally, this pretreatment procedure and therapy are performed in a single setting (1‐day procedure) to minimize changes in anatomy and the catheter position.^19^ Our mobile dual‐layer detector would be moved into the intervention room after the pretreatment procedure to perform an interventional SPECT scan. The activity distribution would be assessed, after which the physician can start the therapy. Our proposed approach to motion compensation is expected to be beneficial for this situation because the time‐critical setting requires fast scans and no inconvenience of an external apparatus.

The purpose of this work is to evaluate the quantitative accuracy and visual quality retrieved from the proposed motion compensation approach for fast interventional liver SPECT in a simulation study. The influence of the fluoroscopic imaging dose, sampling rate, total scanning time, number of bins, and motion patterns will be studied. We will investigate whether inadvertent regions of high activity could be better distinguished with our motion compensation technique than with gating.

## 2 Materials and methods

### 2.1 Detector

**Figure 1:**
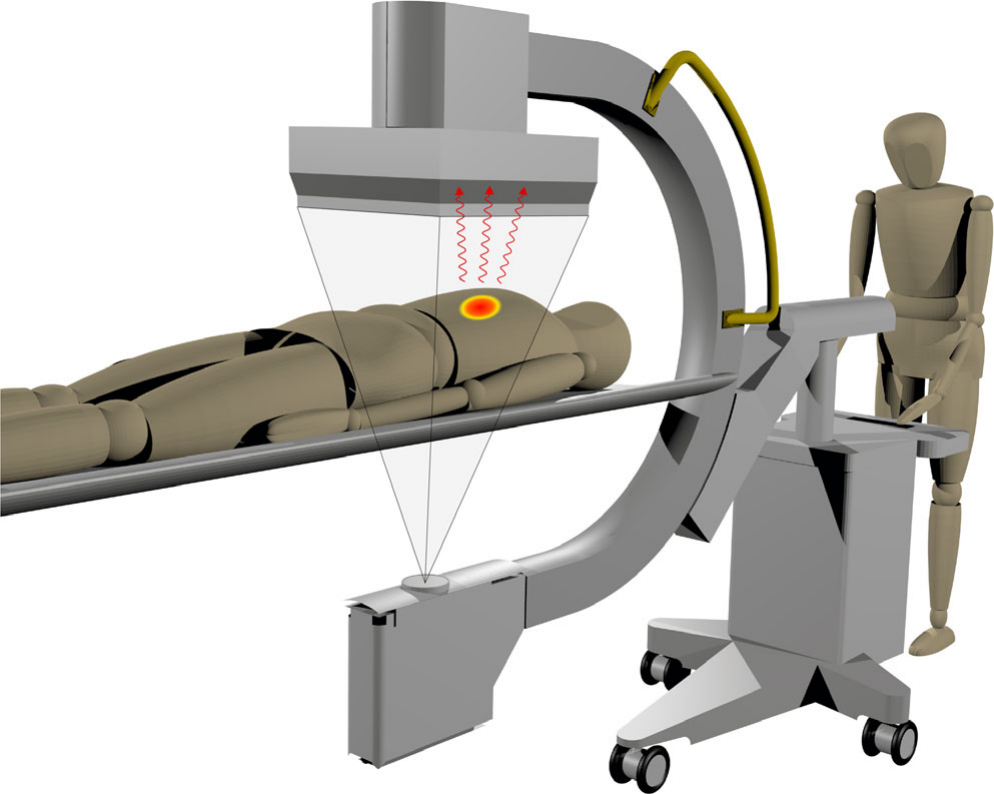
The proposed detector that can measure fluoroscopic and nuclear projections simultaneously. The depicted cone is the irradiated x‐ray area that is captured on the flat panel. In red are the gamma photons that pass through the flat panel to be detected on the gamma camera. [Color figure can be viewed at http://wileyonlinelibrary.com]

An overview of the proposed detector system is shown in Fig. 1. The system consists of a gamma camera with 100‐cm focal length low‐energy, high‐resolution (LEHR) cone beam collimator, merged with a cone beam CT (CBCT) flat panel in front of it. This dual‐layer detector is, together with the x‐ray tube, mounted on a mobile c‐arm so it can be used during interventions. Most generated x rays will be absorbed in the flat panel; the gamma photons possess a higher energy, allowing a major fraction to pass through the flat panel to be detected on the gamma camera. The custom flat panel antiscatter grid was not included because the CBCT reconstruction quality is not of key importance. A prototype system has shown the feasibility of this configuration.^15^, ^16^

### 2.2 Phantom

**Figure 2:**
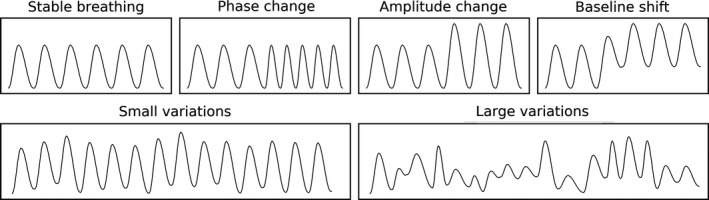
Respiratory motion patterns used in this study: stable breathing and five types of irregular breathing. Visualized is the diaphragm amplitude over time.

A realistic patient phantom was generated using the XCAT phantom program^20^ with the standard body settings of a 95‐kg male. Respiratory motion was generated with 50 samples per breathing cycle, for one stable and five irregular breathing patterns (see Fig. 2).^21^ The six studied patterns were:

1. Stable breathing: respiratory cycle of 5 s with a maximum diaphragm amplitude of 2.0 cm and anterior–posterior (AP) expansion of 1.2 cm
2. Phase change: patient switches halfway in the procedure from stable breathing to a respiratory cycle of 3 s
3. Amplitude change: patient switches halfway from stable breathing to a maximum diaphragm amplitude of 3.0 cm and AP expansion of 1.8 cm
4. Baseline shift: patient switches halfway from stable breathing to a baseline 1.0 cm higher than the previous baseline
5. Small variations: a motion signal with a respiratory cycle of 5 s is generated by randomly assigning a maximum amplitude (between 2.0 and 2.5 cm; scaled AP expansion) and baseline shift (between 0.0 and 0.5 cm).
6. Large variations: a motion signal is generated by randomly assigning a maximum amplitude (between 0.5 and 2.0 cm; scaled AP expansion), phase (between 3 and 7 s), and baseline (between 0.0 and 1.0 cm).

A total of 150 MBq ^99m^Tc was inserted into the liver, in which a sphere of 30 mm in diameter was added. Several uptake ratios (1:2, 1:3, 1:4, and 1:5) were evaluated to study the detectability for the different imaging protocols. Activity and attenuation maps were generated on a 128 × 128 × 100 matrix with 4.7 mm isotropic voxels.

### 2.3 Projection generation

Fluoroscopic projections were generated in GATE,^22^ using the fixed forced detection variance reduction actor for the scatter generation.^23^ Scatter was simulated on a factor 4 downscaled attenuation map, using 5 × 10^5^ particle instances per projection. The input beam spectrum was retrieved from the Simulation of Fluoroscopic Spectra tool^24^ at the clinically used setting of 120 kVp with a 1.0‐mm copper filter. The CBCT flat panel was positioned 27 cm from the body center. The gamma transmission through the flat panel was set at 64%, in line with what we expect to achieve at 140 keV 15.

Nuclear projections were generated using the Utrecht Monte Carlo System (UMCS), which is a fast Monte Carlo‐based software package that simulates particle interactions in the body.^25^ The forward projector has previously been validated for several isotopes^26^, ^27^, ^28^ and has been extended to converging collimators.^29^ An energy window of 15% was set around the 140 keV ^99m^Tc photopeak and the point‐spread function was specifically generated for the proposed detector system, making quantitative reconstructions possible. The intrinsic spatial resolution of the gamma camera was set at 3.8 mm full width at half maximum.^30^ The flat panel thickness (2 cm) increased the nuclear orbit radius to 29 cm. Poisson noise, scaled with the time per projection and the total activity, was added to the projections.

Both fluoroscopic and nuclear projections were generated for 120 angles over 360° for the different breathing patterns at a camera size of 40 cm^2^ × 50 cm^2^. Two protocols were investigated: one with a scan time of 5 min, which will be referred to as the fast interventional protocol, and one with a scan time of 30 min, which is current clinical practice. The fluoroscopic sampling rate was varied from 1 to 5 Hz and the total fluoroscopic dose from 1 to 1000 µGy. Additionally, we investigated whether it would be better to increase the sampling rate or increase the fluoroscopic dose per view, while keeping the total fluoroscopic dose level constant.

### 2.4 Projection noise

To realistically simulate the effect of the fluoroscopic dose on the reconstruction results, a dose–noise relation for the fluoroscopic projections is required. To this end, the noise characteristics of a clinical CBCT scanner (Allura FD20; Philips Healthcare, Amsterdam, the Netherlands) were studied. An image quality phantom (Fluorad A + D; Pehamed, Sulzbach, Germany), consisting of 16 tiles of varying copper thicknesses (representing the range of attenuation in patients), was scanned using a clinical high‐dose CBCT protocol, while monitoring the air kerma reported on the scanner. Additionally, the phantom configuration (as reported in the manual) was simulated in GATE.

The mean values and standard deviations were measured in a square of 74 × 74 pixels positioned on the center of the tiles for a single projection. The fluoroscopic detector noise consists of both a Gaussian (electrical) component and a Poisson distributed component.^31^ The Poisson noise is expected to scale with the square root of the number of detected particles, while the Gaussian noise depends only on the size of the detector pixels. The observed standard deviation for the tiles was therefore fitted with:

$$\sigma =\sigma_{0}+c\;\sqrt{\mu }$$

where *σ* is the standard deviation on the tile, *σ_0_* the electrical noise, *c* a constant, and *μ* the mean value. A change in pixel size results in the scaling of the electrical noise *σ_0_* with the square root of the resize factor and in a linear scaling of the mean value μ.

Using the relation between the mean values of the experimental and simulated (noise‐free) projections and the reported air kerma, the noise in the simulations can be generated as a function of the fluoroscopic dose. In practice, this fluoroscopic dose could be tuned by changing the exposure (mA per projection). Therefore, the fluoroscopic dose would scale linearly with the number of particles detected, thus only influencing the Poisson noise component.

### 2.5 Motion signal extraction

The motion signal was extracted from the pixel intensity distribution of the simulated fluoroscopic projections. Over a vertical strip of 50 pixels, centered on the lung–liver boundary, the center‐of‐mass was calculated for every sampled fluoroscopic projection. The collection of centers‐of‐masses over the projection angles has a background component, which was removed by subtracting the time‐averaged signal. Peaks found in the remaining signal were used to normalize all values between no motion and the maximum motion amplitude. All projections were then binned by placing the bin boundaries at equal distances based on the motion amplitude.

If the number of bins is increased, the resolution of the reconstruction should improve, as more accurate compensation for motion is made. However, the computational time scales linearly with the number of bins and hence an optimum should be found.

### 2.6 CBCT reconstruction

The fluoroscopic projection sets were reconstructed per bin using TIGRE,^32^ which is a fast GPU‐based CBCT reconstruction package with several options for iterative algorithms. The reconstruction was performed using the ordered‐subset simultaneous algebraic reconstruction technique (OS‐SART) with 50 iterations. This iterative reconstruction has an advantage over the regular clinical FDK algorithm^33^ because it suppresses noise.

### 2.7 Registration

The CBCT reconstruction of the first bin was registered to the reconstructions of the other bins using the Elastix image registration package.^34^ The deformable registration provided a motion vector field from the first bin to the other bins. The inverse motion vector fields were retrieved by performing registration with the fixed and moving volumes exchanged, that is, the registration of the CBCT reconstructions of the other bins to that of the first bin. The vector fields were mean filtered over three voxels to increase the smoothness and mean filtered over three motion bins to ensure temporal regularity.

The quality of the CBCT reconstruction is expected to be low (due to a relatively high scatter contribution, small motion artifacts, potential partial sampling, and high noise in the projections). The liver contours themselves may therefore not be tracked over time. Instead, several regions were selected from which to extrapolate the motion. For the superior‐inferior direction, the vector field below the lungs was set to the motion observed in the diaphragm. For the AP direction, the vector field below the lungs was set to the motion observed in the sternum. The spine was assumed to be fixed.

### 2.8 SPECT reconstruction

The nuclear projections were reconstructed using UMCS, with the same settings as in the projection generation. No subsets were used, as the number of projections was not constant for all options. All reconstructions were performed with 25 iterations and 10 noise realizations were made to study the stability of the metrics to noise. Only the dual‐layer configuration was studied in this work, which means that the flat panel was present for all simulated options.

The following options for SPECT reconstruction were compared:

1. No motion compensation. The nuclear projections were reconstructed with no form of motion compensation. This is the current practice in most hospitals.
2. Use of gating. The estimated motion signal was used to select the projections in the breathing cycle with minimum motion: 1/5th of the motion amplitude was used.
3. Use of the motion vector field. For every iteration, during reconstruction and for every bin, the intermediate motion‐free SPECT reconstruction was translated to each respective motion bin using the associated motion vector field. The estimated projections were obtained from the forward projection of the translated reconstruction image, and the error projections were calculated by dividing the estimated projections by the true projections (that should have the same translation due to motion). The error image was obtained by performing backprojection of the error projections. The error images of all bins were translated back to the stationary motion bin, and these images were averaged. This final error image was used to update the intermediate motion‐free SPECT reconstruction, after which the next iteration was started.
4. No motion. A projection set with no motion was generated and reconstructed. These reconstructions were used as a reference.

To correct for photon attenuation, an attenuation map is required. We assumed that a breath‐hold diagnostic CT was available. This high‐quality CT would be registered to the obtained low‐quality CBCT and scaled to generate the breath‐hold attenuation map. For the motion vector field reconstruction option, a 4D attenuation set was subsequently made by applying the respiratory motion transformations.

The incorporation of motion into the SPECT reconstruction was evaluated with two vector fields: one estimated from the motion vector field during the procedure with the proposed detector system, and one that could be obtained from a previously acquired 4D scan. Since motion will vary between scans, the latter vector field was assumed to underestimate the true respiratory motion by 2 mm for the maximum diaphragm motion (resulting in 2.0–0.2 = 1.8 cm max motion) and 1 mm for the AP expansion (resulting in 1.2–0.1 = 1.1 cm max motion) 35.

The reconstruction results were evaluated in terms of the activity recovery (as a measure of quantitative accuracy) and contrast‐to‐noise ratio (CNR) (as a measure of visual quality). The CNR has the additional benefit that it reaches a maximum value, which eliminates the effect of different convergence rates for the several reconstruction methods. Practically, this means that a certain iteration number does not have to be chosen.

## 3 Results

### 3.1 Fluoroscopic dose‐noise relation

**Figure 3:**
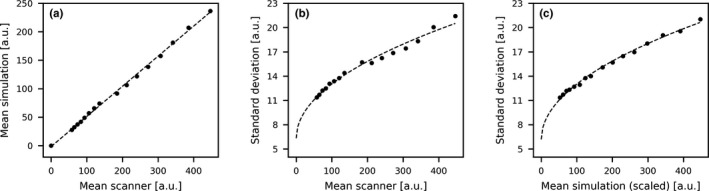
Results on the dose–noise relation in arbitrary units (a.u.): (a) shows the relation between the mean value in the tiles of varying copper thickness observed in the simulation and the scanner projections; (b) shows the standard deviation of the scanner projections as a function of the mean value; and (c) shows that similar noise can be generated on the simulated projections by incorporating the parameters of the scanner fit. The mean values of the simulation were scaled to the mean values of the scanner.

The relation between the mean projection values of the tiles as measured by the clinical CBCT scanner and those simulated with GATE is shown in Fig. 3(a). The relation was found to be linear, which indicates that the simulation is an accurate tool for predicting the scanner projections. Figure 3(b) shows the relation between the standard deviation and the mean values in the scanner projections. Since the curve crosses the x‐origin at a value larger than zero, the electrical noise is important in describing the scanner noise.

With the combined information of the relation between the scanner mean and simulation mean, and the scanner standard deviation, the noise was generated in the simulations. The results are presented in Fig. 3(c), which shows a similar noise pattern as in the scanner projections. This noise is now directly linked to fluoroscopic dose and will be used for further analyses.

### 3.2 Projection generation

**Figure 4:**
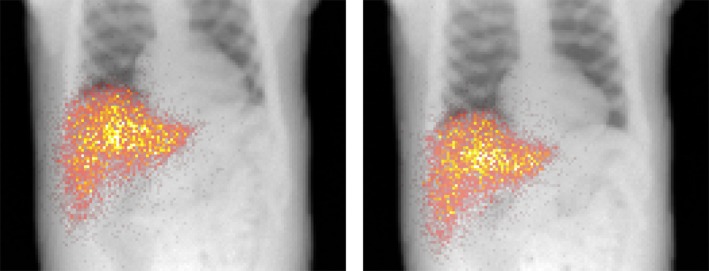
Fluoroscopic (gray scale) and nuclear (color) projections for two respiratory phases (left: amplitude of 0 cm/ right: 5 cm) [Color figure can be viewed at http://wileyonlinelibrary.com]

Two nuclear projections (0.5 s in duration) and corresponding fluoroscopic projections (total fluoroscopic dose level of 1000 µGy) at a diaphragm motion of 0 and 5 cm for the phantom with 1:5 uptake ratio are shown in Fig. 4 for illustration purposes. Histogram equalization was performed on the shown fluoroscopic projections to increase the soft‐tissue contrast for visualization.

**Figure 5:**
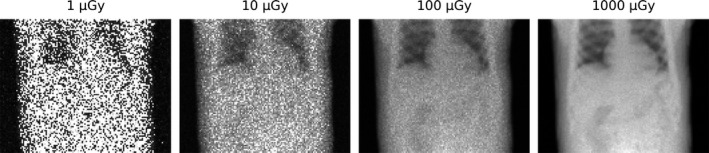
Effect of fluoroscopic dose on the fluoroscopic projections. Reported is the total fluoroscopic dose (air kerma) for all projections in a 5‐min scan while sampling at 5 Hz.

The effect of noise and scatter on the fluoroscopic images can be studied in more detail by evaluating the fluoroscopic projections for the different fluoroscopic dose levels in Fig. 5. It becomes increasingly difficult to identify the individual organs at a lower fluoroscopic dose, but the lung–liver boundary can be visualized even for very low dose levels.

### 3.3 Motion signal extraction

**Figure 6:**
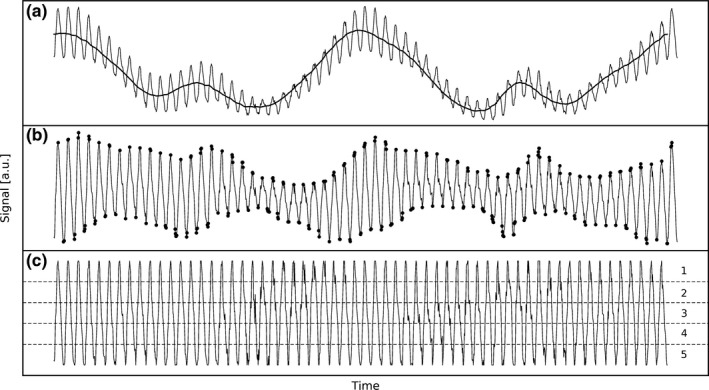
Extraction of the motion signal (acquired with stable motion, a 5‐min scan, a 5 Hz sampling rate, and a 1000 µGy fluoroscopic dose) from the fluoroscopic projections using a center‐of‐mass approach. Frame (a) is the raw motion signal with the time‐averaged signal; (b) the signal with background removed, with the found peaks; and (c) the normalized signal with five bin boundaries.

Figure 6 shows the motion signal that was extracted from the fluoroscopic projections for a stable respiratory pattern (acquired with 1000 µGy fluoroscopic dose, 5 Hz sampling rate, and 5‐min scan). Figure 6(a) shows the raw signal obtained from the center‐of‐mass tracking, together with the time‐averaged signal. Figure 6(b) shows the signal with the background component removed (i.e., the raw signal minus the time average), together with the found peaks. Figure 6(c) shows the signal normalized to the peaks, together with the bin boundaries.

### 3.4 CBCT reconstruction

**Figure 7:**
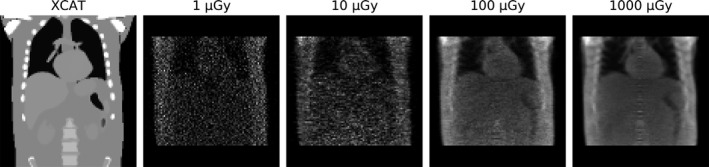
Reconstructions of one bin for the studied fluoroscopic dose levels (acquired with stable motion, a 5‐min scan, and a 5 Hz sampling rate), together with the XCAT phantom used as input for the fluoroscopic projections.

The binned fluoroscopic projections were reconstructed per bin, and the result for one bin is shown in Fig. 7. Some artifacts toward the ribs are present due to intra‐bin motion. The CBCT reconstruction image contrast was lower than that of the XCAT phantom due to beam hardening and the contribution of scattered photons. In the upper and lower parts of the reconstruction, some volume is truncated because of the scanner size and the cone geometry. Again, it is difficult to localize the individual organs, but the lung–liver barrier can be well identified.

### 3.5 Motion vector field

**Figure 8:**
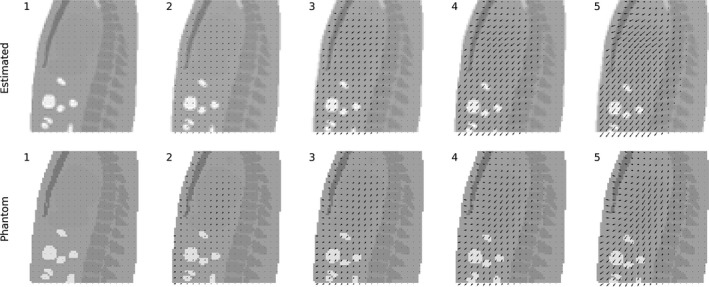
Estimated motion vector field for five bins (acquired with stable motion, a 5‐min scan, a 5 Hz sampling rate, and a 1000 µGy fluoroscopic dose) with the transformed attenuation map (top) and the phantom motion field with the phantom attenuation map (bottom).

The CBCT reconstructions of the binned fluoroscopic projections were coregistered, from which the motion field was extracted. Figure 8 shows the evolution of the motion field over five bins, together with the motion field of the phantom. The motion in both the superior‐inferior and AP directions is evident. The abdomen is enlarged in size in the transformed attenuation map over the respiratory cycle.

### 3.6 SPECT reconstruction

**Figure 9:**
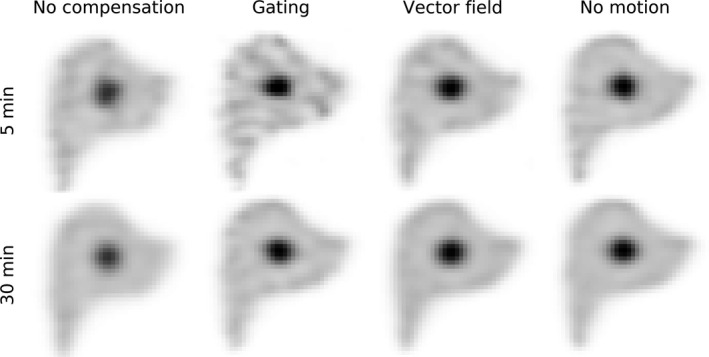
Reconstructions of the nuclear liver images for stable motion for a single noise realization, with both the five‐ and 30‐min scans for the phantom with a 1:5 uptake ratio. Reconstruction was performed without motion compensation, with gating, and with vector field compensation (acquired with a 5 Hz sampling rate and a 1000 or 6000 µGy fluoroscopic dose). As a reference, the last column shows the reconstructions for the case in which no motion was present. The iteration in which the maximum CNR was obtained is shown.

Figure 9 shows the SPECT reconstructions for the four reconstruction methods for both the 5‐ and 30‐min scans (acquired with stable motion, 5 Hz sampling rate, 5 bins, and 1000 µGy (5‐min) or 6000 µGy (30‐min) fluoroscopic dose). Visual inspection shows that no motion compensation resulted in a low contrast between the sphere and liver background. Gating resulted in a high‐contrast image, but the noise in the background was increased, which was especially evident in the 5‐min scan. Motion compensation resulted in a high sphere contrast and low noise levels. This option best resembled the reconstructions with no motion present.

**Figure 10:**
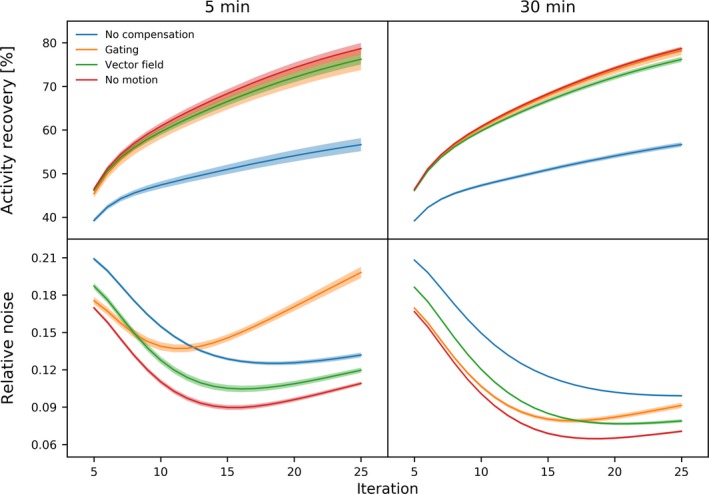
Sphere activity recovery and relative background noise for the motion compensation options, shown for the 5‐ and 30‐min scans (acquired with stable motion, a 5 Hz sampling rate, and a 1000 or 6000 µGy fluoroscopic dose) for the phantom with a 1:5 uptake ratio as a function of the iteration number. The shaded regions represent the standard deviation obtained from the noise realizations. [Color figure can be viewed at http://wileyonlinelibrary.com]

The above results are presented in more detail in Fig. 10, which shows the activity recovery and the relative noise levels (standard deviation divided by the average intensity) as a function of the iteration number. These results confirm that both gating and the vector motion field compensation provided good quantitative accuracy, but noise quickly dominated in the case of gating.

**Figure 11:**
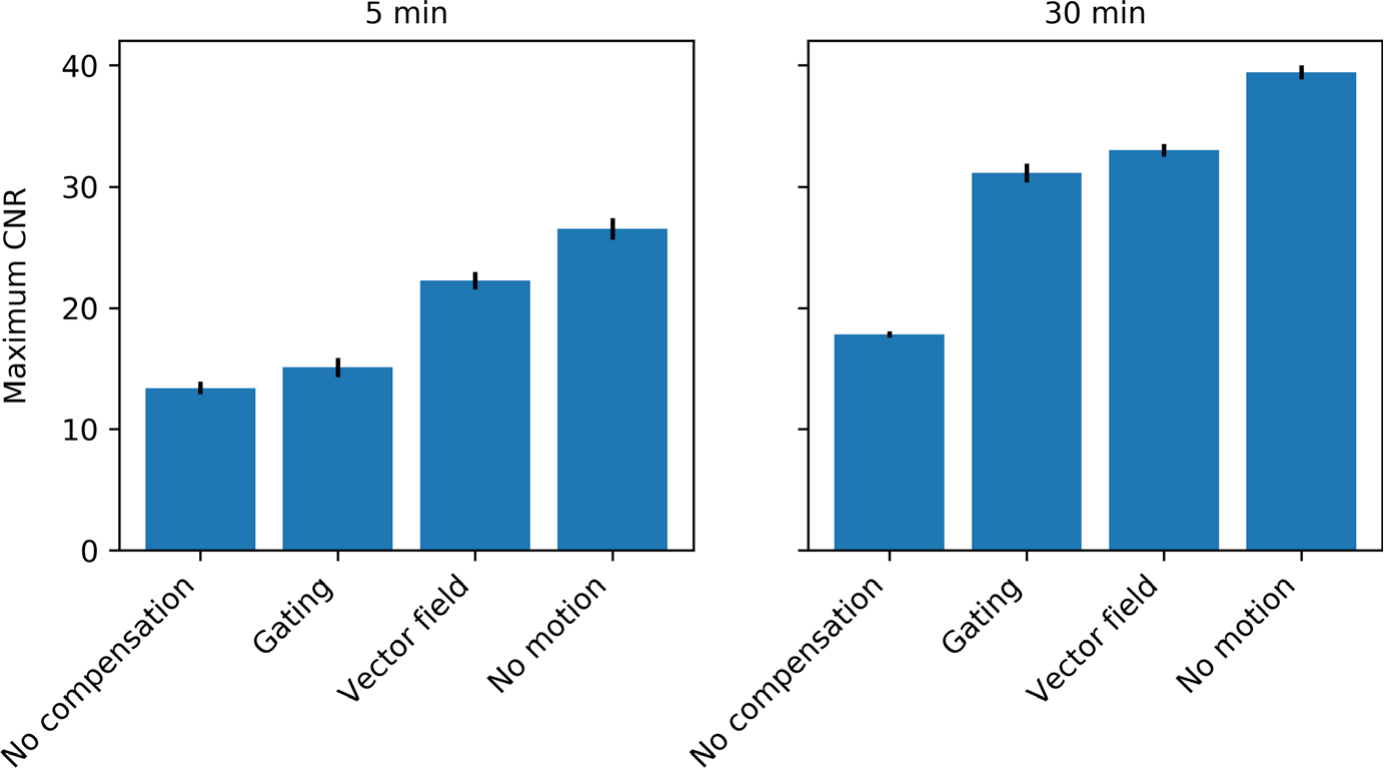
Maximum CNR of the sphere in the SPECT reconstruction for the various reconstruction methods, for the 5‐ and 30‐min scans (acquired with stable motion, a 5 Hz sampling rate, and a 1000 or 6000 µGy fluoroscopic dose) for the phantom with a 1:5 uptake ratio. [Color figure can be viewed at http://wileyonlinelibrary.com]

Using the contrast‐to‐noise ratio, the above results can be combined in a single measure. The maximum CNR over the iterations was determined and is shown in Fig. 11. This figure shows how gating performed relatively better for the 30‐min scan than for the 5‐min scan, thanks to the increased number of counts available for reconstruction.

### 3.7 Reconstruction properties

The maximum CNR for the studied reconstruction properties for the 5‐min scan are shown in Table 1. The standard vector field compensation is the reconstruction performed with 1:5 uptake ratio, stable motion, 5 Hz sampling rate, 1000 µGy fluoroscopic dose, and five bins. The studied reconstruction options have settings equal to those of this standard vector field compensation unless specified otherwise.

**Table 1:** Maximum CNR values for the several reconstruction options for the 5‐min scan. No compensation resulted in a maximum CNR of 13.4 ± 0.4. No motion resulted in a maximum CNR of 26.5 ± 0.9.

Number of bins	CNR	Fluoroscopic dose	CNR	Sampling rate	CNR
2 bins	21.4 ± 0.6	1 µGy	16.8 ± 1.2	1 Hz	22.0 ± 0.9
3 bins	21.4 ± 0.7	10 µGy	21.1 ± 0.7	2 Hz	22.6 ± 1.0
5 bins	22.3 ± 0.7	100 µGy	22.3 ± 0.8	3 Hz	22.4 ± 1.3
10 bins	22.4 ± 0.8	1000 µGy	22.3 ± 0.7	5 Hz	21.1 ± 0.7

We investigated the cases of 2, 3, 5, and 10 bins. Even for only two bins, the CNR was boosted significantly in comparison with no motion compensation. Using additional bins improved the CNR, but this increase was much smaller. This is because the magnitude of motion (average diaphragm amplitude of 10 mm for stable breathing) is in the same order of magnitude as the resolution of the gamma camera (~7.5 mm at 10 cm) 30. Accordingly, the mismatch in time‐averaged nuclear data with snap‐shot fluoroscopic projections does not significantly influence the reconstruction quality.

The fluoroscopic dose levels were varied from 1 to 1000 µGy. A fluoroscopic dose of 10 µGy boosted the CNR considerably compared to 1 µGy dose. However, an increase in the fluoroscopic dose did not further substantially improve the results.

The x‐ray sampling rate was varied between 1, 2, 3, and 5 Hz at a total constant fluoroscopic dose level of 10 µGy. This fluoroscopic dose level was chosen, as it was shown that this is the threshold for vector field motion compensation to function. The highest CNR was achieved at a fluoroscopic sampling rate of 2 Hz. Fluoroscopic noise started to dominate with a further increase in the sampling rate, thus decreasing the CNR.

The effect of using a motion vector field from a previously acquired 4D scan (with an inaccuracy of a few mm in the motion amplitudes), instead of using the vector field from the scan of interest, resulted in a CNR of 21.5 ± 0.7 instead of 22.3 ± 0.7.

The effects of irregular breathing motion were evaluated by performing reconstruction with several breathing patterns. These results are shown in Table 2. The reconstruction quality was largely unaffected by phase changes. The reconstruction quality increasingly deteriorated for amplitude changes, small variations, and baseline shifts. Vector field motion compensation led to minimal improvements in the reconstruction quality for large motion pattern variations, but yet it did not reduce performance more than when no motion compensation was applied. This suggests that the proposed motion compensation can always be performed since reconstruction quality did not decrease in the studied situations.

**Table 2:** Maximum CNR values for the irregular breathing patterns for the 5‐min scan for no compensation, gating, and use of the motion vector field. No motion resulted in a maximum CNR of 26.5 ± 0.9.

Breathing pattern	No compensation	Gating	Vector field
Stable	13.4 ± 0.4	15.1 ± 0.8	22.3 ± 0.7
Phase change	13.7 ± 0.7	15.4 ± 1.0	21.8 ± 0.9
Amplitude change	11.6 ± 0.6	15.5 ± 0.7	20.7 ± 0.9
Baseline shift	8.8 ± 0.3	12.7 ± 0.7	17.7 ± 0.7
Small variations	8.6 ± 0.4	14.0 ± 1.0	18.5 ± 0.7
Large variations	11.0 ± 0.4	10.4 ± 0.7	13.3 ± 0.7

Finally, the difference in the detectability of regions with high activity using the two motion compensation methods was evaluated by reconstructing phantoms with different uptake ratios. These results are shown in Table 3. If the level of detectability is set at the Rose criterion of a CNR level of 4,^36^ the spheres with 1:2 uptake ratio would not be detected using gating. Conversely, this sphere could be observed using motion vector field compensation.

**Table 3:** Maximum CNR values for the spheres with varied uptake ratios for the 5‐min scan for no compensation, gating, and use of the motion vector field.

Uptake ratio	No compensation	Gating	Vector field
1:2	2.7 ± 0.3	3.2 ± 0.4	4.4 ± 0.5
1:3	6.4 ± 0.4	7.1 ± 0.6	9.9 ± 0.5
1:4	10.0 ± 0.5	11.0 ± 1.0	15.7 ± 1.2
1:5	13.4 ± 0.5	15.1 ± 0.8	22.3 ± 0.7

## 4 Discussion

In this study, we evaluated whether motion compensation using simultaneous fluoroscopic and nuclear imaging could enhance the SPECT quality in fast interventional liver scanning.

In hepatic radioembolization, ^99m^Tc‐MAA SPECT/CT imaging is performed for the detection of inadvertent regions of high activity and for the treatment planning of dosimetry. We have shown that regions with low uptake ratios can be better detected with our motion compensation method than with gating. This argument similarly holds for regions of increasingly smaller sizes. We acknowledge that high visual quality is currently not crucial for dosimetry, as activity recovery remains accurate for low count rates as with gating.^37^ However, it could be argued that uniformity will become an increasingly important measure when voxel‐based dosimetry^38^ is further developed, as the activity will then also need to be correctly measured for small regions.

Two limitations of this study were that a phantom with rigid‐like liver motion was evaluated and that the proposed method assumed a non‐deformable liver. The method discussed in this work is furthermore heavily dependent on the visibility of the lung–liver barrier to extract the motion vector field. Assumptions have been made on the liver displacement in relation to the other organs, the validity of which will be studied in future work. For now, the effect of motion compensation should be carefully monitored in the case of regions of high activity further away from the liver. While the current implementation of the motion vector field requires a manual selection of regions to extrapolate the lung motion to the liver motion, this step should be automated once the system has passed the prototype phase. This is expected to be feasible because the boundaries between lung, air, and soft tissues are well discernible.

The background noise in gating could, to some extent, be compensated for in the reconstruction using regularizers, such as total variation minimization.^39^ The smoothing of uniform regions, while preserving sharp edges, would improve the results on CNR similarly to the proposed method. However, since activity distributions will vary substantially between patients, the best setting for the required regularization parameter is often not evident. The use of optimal gating.^40^ could similarly provide smoother reconstructions but will come at the cost of a loss in resolution.

The prototype dual‐layer detector system^15^, ^16^ is currently being integrated into a custom C‐arm. In the present simulation study, it was assumed that the flat panel could be decreased in thickness and attenuation by relocating the detector electronics. The camera size was additionally increased, and the detector orbit was reduced so that a closer orbit around the patient could be realized. X‐ray overflow on the gamma camera was not considered since this is not a limiting factor at low fluoroscopic doses.^41^, ^42^

The simultaneous anatomic and nuclear information needed to acquire a motion vector field might also be successfully obtained using integrated SPECT/MRI,^43^ which does not have the downside of delivering radiation doses to patients and might better cope with irregular breathing. The main benefit of the proposed detector is that it is relatively small and mobile, and thus suited for interventional procedures. Additionally, by designing the gamma camera as an add‐on to a regular c‐arm, the manufacturing cost can be kept low and implementation in the clinic can be accelerated.

A radiation dose is required for our approach to motion compensation. However, it is not expected that this will limit clinical use. First, a radiation dose will always be required for interventional scanning, inasmuch as the SPECT scan needs either a self‐recorded attenuation map or a volume to perform registration on. The ability to perform motion compensation can thus be considered an added benefit. Second, it was shown that our approach required only 10 µGy for the described situation. This is very low in comparison with, for example, the generation of an attenuation map in SPECT, which is usually in the order of several mGy 44. And third, in the proposed application of hepatic radioembolization, patients locally receive several hundred Gy, many orders of magnitude more than in our approach.

Breathing motion patterns consist of a combination of phase changes (breathing faster or slower), amplitude changes (breathing more/less deeply), and baseline shifts (switching of breathing pattern).^45^ Algorithms, such as the Amsterdam Shroud,^46^ Fourier‐based approaches,^47^ and principle component analysis,^48^ cannot determine these amplitude and baseline changes. Furthermore, approaches using template matching^49^, ^50^ require high‐contrast images, which are not available if the administered fluoroscopic dose is kept low.

It was shown that changes in respiratory motion degrade the reconstruction results. However, the extent to which such motion variations will be present in an interventional (e.g., radioembolization) setting is unclear, as we unfortunately do not have access to patient respiratory signals. Patients are generally already on the operating table for some time before the scout scan is performed, which might reduce the likelihood of a change from an active to a resting state. Additionally, patients are often sedated to some degree, which will reduce stress‐related breathing. For now, we assume that the motion vector field can be approximated as stable during a single scan. Future studies are needed to determine whether this assumption holds.

The prototype as described performs a single rotation only and is thus subject to the limited angle effect since projections are only available under certain angles.^51^ One way to better cope with the influence of irregular breathing would be to perform multiple rotations during the scan. Faster rotation, however, requires better mechanical stability, which may not be achieved in the prototype under construction. This option has therefore not been further explored in this study but could be beneficial for future designs.

As discussed previously, an approximation of the motion vector field could be estimated with other modalities, such as CT or MRI. A major advantage of our motion compensation approach is that it is not sensitive to potential changes in breathing between scans, making it a reliable option for clinical practice. The above options would also require that the previously acquired 4D scan be matched to the SPECT scan, requiring a registration step. This registration could result in blurring, which is an additional disadvantage of using the vector field from a previously acquired 4D scan. The integrated SPECT/CBCT system captures the same field of view and hence does not have this problem.

By assigning nuclear counts to gates using the motion signal from an external gating device, it might be possible to estimate the motion vector field from the resulting reconstructed SPECT volumes. This approach has the benefit of not requiring any other modality for its motion compensation, and thus not requiring any hardware modifications. However, for fast scanning, we expect that the quality of the SPECT volumes will be severely limited by counting statistics, making it very challenging to estimate a correct vector field. While this approach might thus be beneficial for general motion compensation in SPECT, it is unlikely to succeed in a time‐critical interventional setting.

It is not expected that the use of an antiscatter grid will improve the detection of the lung–liver barrier since the difference in density between these tissues is relatively large. However, when simultaneously applying a higher fluoroscopic dose, the soft‐tissue contrasts in the reconstructions may become more apparent. Tracking all organs individually may then still be challenging but should be feasible with the use of dedicated registration software. Such an approach would additionally allow deformable registration, in contrast to the proposed method, which assumes a rigid liver. Our aim is to limit the fluoroscopic dose rate initially and evaluate its performance. If it proves that the used extrapolation method does not accurately describe the respiratory motion, the option above could be further explored.

Three steps in the total reconstruction process take up most of the reconstruction time. For a single bin, and with a single thread on a regular desktop computer, the required times are approximately 10 s for CBCT reconstruction, 2 min for registration, and 30 min for SPECT reconstruction (for 25 iterations). All bins can be processed in parallel, which means that the total number of bins will generally not be limiting. With code optimization and the use of dedicated hardware, we expect that a total reconstruction time of 5 min, fast enough for use in an interventional setting, can be achieved.

## 5 Conclusion

We have developed and evaluated a motion compensation approach using simultaneous fluoroscopic and nuclear imaging with a dual‐layer detector. Such a configuration can intrinsically measure the respiratory motion signal and the associated motion vector field during a SPECT scan, which eliminates the need for external devices and provides an optimal motion vector field. The SPECT reconstruction quantitative accuracy is boosted substantially compared with no motion correction; the visual quality is also improved compared to gating. Combined with the fast reconstruction implementation, this will allow for motion‐corrected scans in the intervention room.

## Disclosure

This project has received funding from the European Union’s Horizon 2020 Research and Innovation Program under grant agreement No 646734. No other potential conflicts of interest relevant to this article exist.
